# Monitoring, evaluation and accountability evidence use for design, adaptation, and scale-up of an early childhood development program in Rwanda

**DOI:** 10.3389/fpubh.2023.1165353

**Published:** 2023-07-27

**Authors:** Caroline Dusabe, Monique Abimpaye, Noella Kabarungi, Marie Diane Uwamahoro

**Affiliations:** ^1^Save the Children Australia, Melbourne, VIC, Australia; ^2^Save the Children (Rwanda), Kigali, Rwanda

**Keywords:** early childhood development, parenting, measurement, monitoring, evaluation, program adaptation, scale-up, evidence

## Abstract

**Introduction:**

The first three years of a child’s life are the most critical to child development and have an impact on the future achievement of the child. Young children’s healthy development depends on nurturing care that ensures health, nutrition, responsive caregiving, safety, and security. Parents & other adult caregivers play a critical role in moderating children’s early experiences, which has a lasting impact be it positive or negative on the children’s future. Parenting education programs are proven to improve parental skills, capacity, and efficacy in a way that supports improved child development outcomes. Yet, most parents in low-middle-income countries such as Rwanda lack access to information and skills on how to support their children’s holistic development. In response, Save The Children implemented the First Steps “Intera za Mbere” holistic parenting education project in Rwanda from 2014 to 2021. This paper reflects on how monitoring, evaluation, accountability, and learning (MEAL) approaches were applied throughout the project cycle and their impact on program improvement and national policy and advocacy. This paper explores how the aspirations for measurement for change, considerations for innovation uptake and frameworks for learning about improvement are reflected in this project.

**Methods:**

The project utilized qualitative and quantitative MEAL across the program cycle. Action research at the start of the project identified promoters and inhibitors of high-quality nurturing care and program delivery modalities. The project utilized a randomized control trial to provide insight into components that work better for parenting education. Evidence from surveys done remotely via phones was used to inform COVID-19 adaptations of the program.

**Results:**

The application of MEAL evidence led to the successful development and improvement of the program. At the policy level, evidence from the project influenced the review of the 2016 National Integrated ECD policy and the development of the national parenting education framework.

**Conclusion:**

The regular use of evidence from MEAL is critical for program improvement, scale-up, and policy influence.

## 1. Introduction

Globally more than 250 million children are not meeting their developmental potential due to a myriad of factors including lack of stimulation, poor health, and nutrition, exposure to violence in their homes, and humanitarian crisis (1). The first 3 years of a child’s life are critical for brain development, physical development, and forming social and emotional skills (2). Young children, therefore, need a nurturing environment that comprises early learning, child protection, responsive caring, health, and nutrition quality services, and parents should be equipped to provide quality holistic ECD services to their children (3).

In Rwanda, the nutrition and development status of a child has been improving over the last 6 years but more still needs to be done. When the First Steps Intera za Mbere program started in 2014, the stunting rate in Rwanda was at 38% and this has now reduced to 33% as per the 2020 Rwanda Demographic and Health Survey (RDHS). But, young children in Rwanda are still faced with many challenges including limited stimulation and early learning support at home. In the RDHS 76% of children aged 24 to 59 months were found to be developmentally on track in at least 3 of 4 core domains of child development although only 12% were found to be on track in literacy and mathematics, which are foundation skills for later learning. Children living in urban areas and children from wealthier households were found to be more likely to be on track implying issues around equity. In terms of access to play and learning materials, only 2% of children under 5 were found to have 3 or more picture books at home, and only 36% of children aged 0–59 months had engaged with an adult household member in four or more activities that promote learning and school readiness 3 days prior to the survey (4, 5). This indicates low stimulation and opportunities for learning in the home environment.

In response to these challenges, in 2014, The First Steps “Intera za Mbere” (FS) project was innovated, designed, and implemented in the western province district of Ngororero. In 2018 it was scaled up to 3 more districts, Kirehe, Gasabo, and Ruhango. The project aimed at offering a nationally scalable and holistic approach to parenting education that was integrated within the Rwandan government structures, combining radio programming with community-based peer learning groups, and collaborating with local publishers and entrepreneurs to increase parents’ access to emergent literacy materials. The project also had an ambitious policy and advocacy agenda to ensure that the enabling environment for nurturing care was improved and sustained through favorable policy actions. The ambitions for scale and sustainability required merging learning and implementation science approaches to capture what works or does not work, how it works and other factors necessary for innovation uptake as proposed by Danika (6) and Bauer et al. (7) and other implementation scientists exploring the systematic uptake of research findings and other evidence-based practices (EBPs) into larger systems.

## 2. Context

Prior to 2014, the focus on the National 0–3 Early Childhood Development (ECD) agenda was limited to mainly health and nutrition. The 2011 Early Childhood Development Policy emphasized early learning for children above 3 years, with limited to no recognition of cognitive development and early learning from birth (8). Advocacy including from Save the Children led to a shift to holistic development for the 0–3 age with a focus on early learning and stimulation. The latest 2016 National Early Childhood Development policy overseen by the Ministry of Gender and Family Promotion (MIGEPROF) recommended interventions that support a child’s whole development from 0 to 6, with parenting education as a key pillar. The government has since then accelerated efforts to implement the policy in collaboration with partners.^1^ Save the Children has positioned the First Steps intervention as a source of learning and evidence on what works best to implement the parenting education pillar of the policy. The target population for First Steps is expectant parents and parents with children aged 0–36 months.

## 3. Key programmatic elements

First Steps “Intera za Mbere” is a radio-supported holistic parenting education program targeting male and female caregivers of children aged 0–3 years old. The program aims to improve nurturing care practices, child development, and learning outcomes, and increase emergent literacy promotion in the home. Typically, parents attend 18 structured 60–90-min parent group sessions run by a trained parent facilitator. Seventeen of the 18 sessions include a radio listening session which is preceded and followed by facilitated discussion, reflection, demonstration, and practice of key nurturing care behaviors such as singing, reading, talking, telling stories, and playing with children. Parents are requested to attend the session together with their children which gives them an opportunity to play and practice simple games that support children’s development.

A distinct aspect of the radio program is that it addresses male engagement through the role model of a father whose character evolves over the course of the drama, from a skeptic into a loving, nurturing, playful father. A light touch home visit component was also piloted in the project^2^. The project was implemented from 2014 to 2021 by Save the Children and a local Non-Governmental Organization (NGO) implementing partner Umuhuza. In 2022, the project underwent iteration to focus on system strengthening at scale as discussed below. The First Steps project was implemented in 4 districts, however, as part of the scale-up strategies NGO partners such as Help a Child and A Partner in Education (APIE) were given access to the parenting education tools for use in other locations. The radio program was broadcast throughout the whole country during the COVID-19 period when face-to-face gatherings were limited. So, this means that some program elements have scaled nationally.

On the journey to scale, First Steps utilized monitoring, evaluation, accountability, and learning (MEAL) approaches, and human-centered design principles to allow evidence and learning from program implementation and evaluation to shape every stage of the program cycle. Evidence and learning from the program were also used to influence national-level policy, practice, and advocacy. Both qualitative and quantitative methods were used to gather program learning and evidence. Over 6 years, the program went through several distinct phases punctuated by periodic “pause-reflect-action” cycles that allowed the program to continually apply measurement for change principles and aspirations. The distinct phases and key activities are discussed below.

### 3.1. Action research and human-centered design- 2014

First Steps was launched in Ngororero District of Rwanda in 2014 and the first phase was devoted to action research, engaging community members in identifying promoters and inhibitors of high-quality nurturing care among parents of children aged 0–3. Parents and parent trainers also provided feedback on the appropriate materials for training, length of training, home visits, and other program inputs. Initially, two blended curricula, “Child I Care” from Umuhuza and First Read 0–3 from Save the Children were used to pilot and learn what works best in parenting education in this setting. “Child I Care” emphasized social–emotional development while First Read had an emphasis on early learning and stimulation. The two curricula were blended to form a 13-week-long pilot parenting program. The purpose of this phase was to learn from parents and parent facilitators what worked well or did not work in terms of the delivery of parent sessions, materials needed, and relevance of topics to parents’ needs. Volunteer parent facilitators managed by Umuhuza carried out parenting education sessions and home visits weekly and documented their experiences. The facilitators had reflection forms with guiding questions that they had to fill out after every group session and a home visit. In addition, the Save the Children ECD technical lead together with MEAL personnel carried out session observations and led bi-weekly facilitators’ group reflection. Two cohorts of pilot parent group sessions were run with 800 families in two sectors of the Ngororero district. In addition, focus group discussions were held with both parents from the pilot sites and parents from other sectors not yet reached with parenting messages to understand the existing nurturing care practices, gaps in nurturing care among both male and female caregivers, as well as preferred modalities of receiving parenting messages. Both parents and facilitators recommended having one harmonized curriculum and they recommended priority topics based on what they understood to be gaps in their parenting knowledge. They also recommended having visual posters for parent group sessions and printed take-home materials. Through community consultations, the design team also noted the high interest in soap opera radio drama for passing social behavior change messages. Parents noted that they enjoyed an existing popular soap opera called Urunana. Parent facilitators provided feedback on home visits and suggested a reduction of required home visits per family to reduce the burden on volunteers. At the national level, a political economy analysis (PEA) and stakeholder listening session done as part of a larger 0–9 Advancing the Right to Read PEA study conducted by Save the Children, also provided insights into enabling factors that were needed for success including an ECD policy revision with emphasis on the thus far neglected 0–3 age range and challenges of 0–3 ECD front line workforce.

Based on this learning and feedback, a holistic parenting education curriculum and training materials including a 17-session radio soap opera program were developed. The radio program served a dual purpose of passing messages to parents while also reducing message dilution that normally comes due to cascade training modalities such as what was being used in the program. In early 2015 the new First Steps “Intera za Mbere” curriculum and materials were tested, refined, and validated. Feedback from the field in Ngororero was combined with feedback from national level stakeholder consultation to refine the program tools for the proof-of-concept phase. Save the Children also began intensive policy and advocacy on investment in parenting education and support for parents of children birth to 6 years. Advocacy and policy materials including a strategy and a parenting education advocacy brief were developed and utilized at all levels of change from local to national level.^3^ This phase was marked by all Measurement for Change (M4C)^4^ aspirations especially being people-centered and inclusive.

### 3.2. Proof of concept phase, 2015–2016

From 2015 to 2016, the program entered a proof-of-concept phase focused on determining the most feasible, cost-effective, and scalable approach to delivering parenting education. A Randomized Control Trial (RCT) was conducted with two modalities of interventions and a control group; *Light intervention* characterized by: 3.5-day training for a local volunteer, a basic package of materials,^5^ and parenting education sessions supported by radio; *Full intervention* characterized by all light touch inputs, plus the provision of an enhanced package of materials^6^ and additional training for the volunteer on how to use them; plus a salaried area facilitator supporting the local volunteers in guiding group sessions and conducting home visits; *Control group* receiving no support during the active implementation period. However, the control group received the light intervention package after the research period concluded. The RCT baseline confirmed the urgent need for investments in parenting education and early learning interventions from birth as children showed a declining ability to meet age-appropriate developmental benchmarks as measured by the Ages and Stages Questionnaire (ASQ), especially for communication and problem-solving which are predictive of later academic success. The end-line results revealed that children in the intervention group outperformed their peers in the control area. The evaluation also found that the frequency and quality of parental play and learning activities were correlated with children’s developmental outcomes.^7^ In addition, to the RCT, Save the Children continued to collect data on the implementation to understand what was working or not working in program delivery. Learning from this phase was used to adjust the delivery model including the shift from utilizing trained parents as volunteer parent facilitators to utilizing the newly formed government cadre of social worker volunteers called Inshuti z’Umuryango (IZUs) /Friends of the family commonly known as IZUs^8^. Adaptations were also made to the peer coaching and mentorship model and required home visits per family were reduced to two per family in a 17-week period to match facilitator workload demands. A practical session on cooking demonstration was also added as per demand from both parents and facilitators. The curriculum was also updated further to add more age and culturally-appropriate games for children aged 0–3 years and increase the understanding of community volunteers on 0–3 playing activities to include during the face-to-face parenting session. Beyond Rwanda, the learning from the project was shared within the Save the Children movement and contributed to the development & refinement of Save the Children’s 0–3 Building Brains Common Approach.^9^

### 3.3. Transition to scale (TtS), 2017–2019

In 2018, a follow-up RCT measured the medium-term impact of the intervention on children and parental outcomes 24 months after the intervention. Again, parents and children who benefited from the intervention continued to outperform their peers from the control group suggesting the midterm to long-term impact of the intervention on parental practices and children’s developmental outcomes.^10^ Implementation research data was collected to inform national scaling-up plans. A component of the follow-up study was to understand the benefit of sharing evaluation results with parents via accessible video media. Through this follow-up study, the team learned about the value of the radio program as parents continued to rank radio listening as one of the most important elements of the intervention. The team also learned about the benefits of sharing results with the parents in increasing parental confidence and efficacy. When parents heard that their simple actions such as singing and talking were leading to improved outcomes for children, they did those actions even more. However, the results from this study also revealed that caregivers were not making strong gains in applying positive discipline strategies in comparison to the application of strategies that support early learning, nutrition, and health. Therefore, the team made a decision to once more update the curriculum to mainstream positive discipline into all 17 structured sessions in addition to maintaining the topic as a stand-alone. Practical and age-appropriate positive parenting messages and strategies were shared at every parent group meeting, as well as gender messages to emphasize the role of both parents in child development. The detailed results from the follow-up RCT are shared in another paper (12).

First Steps entered the transition to scale in mid-2019. The 24 months long TtS phase intended to test if the program could be implemented in new geographical areas (including both rural and urban settings) with similar gains and to collect implementation science data to inform national scale-up plans. The program was implemented in Ruhango, Kirehe, and Gasabo Districts in October 2019 with minor modifications to the treatment conditions of the previous phase and following an RCT design. The parenting sessions, home visits, and parent support activities were carried out by IZUs, in line with learning from previous phases. Working with this existing government structure was one of the sustainability strategies of the program. During this period, as part of scaling strategies, *via* a signed Memorandum of Understanding (MoU), Save the Children also shared the technical tools with NGO partners Help a Child and A Partner in Education to implement within their own programs. These partners met annually with Save the Children and Umuhuza to reflect and share learning from implementation and contribute to decisions on the program improvement. This phase especially exemplified the M4C aspiration of being dynamic and informative.

### 3.4. COVID-19 adaptation and national scale up, 2020–2021

**Figure 1:**
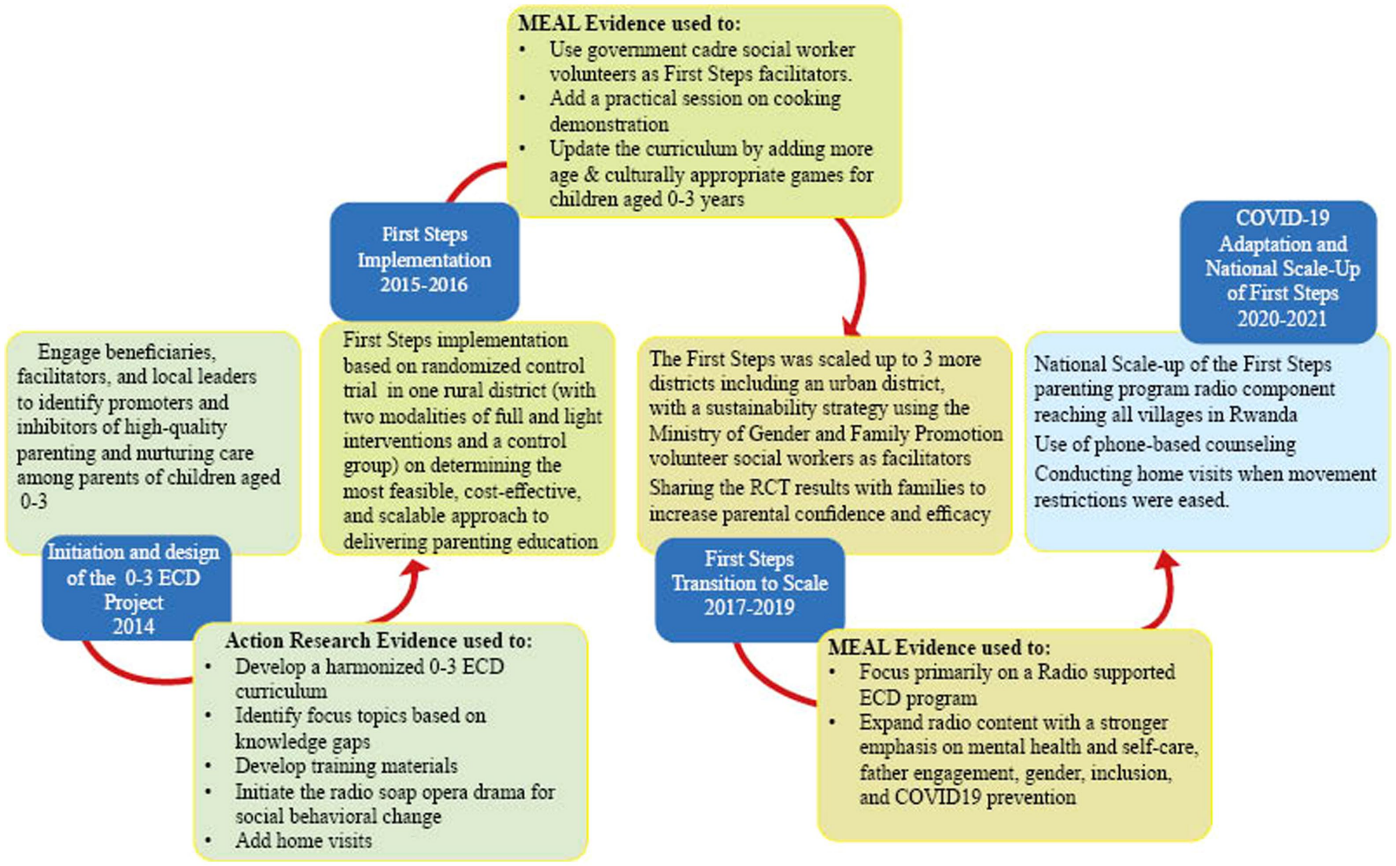
First steps “Intera za Mbere” measurement for change loops and decisions.

**Figure 2:**
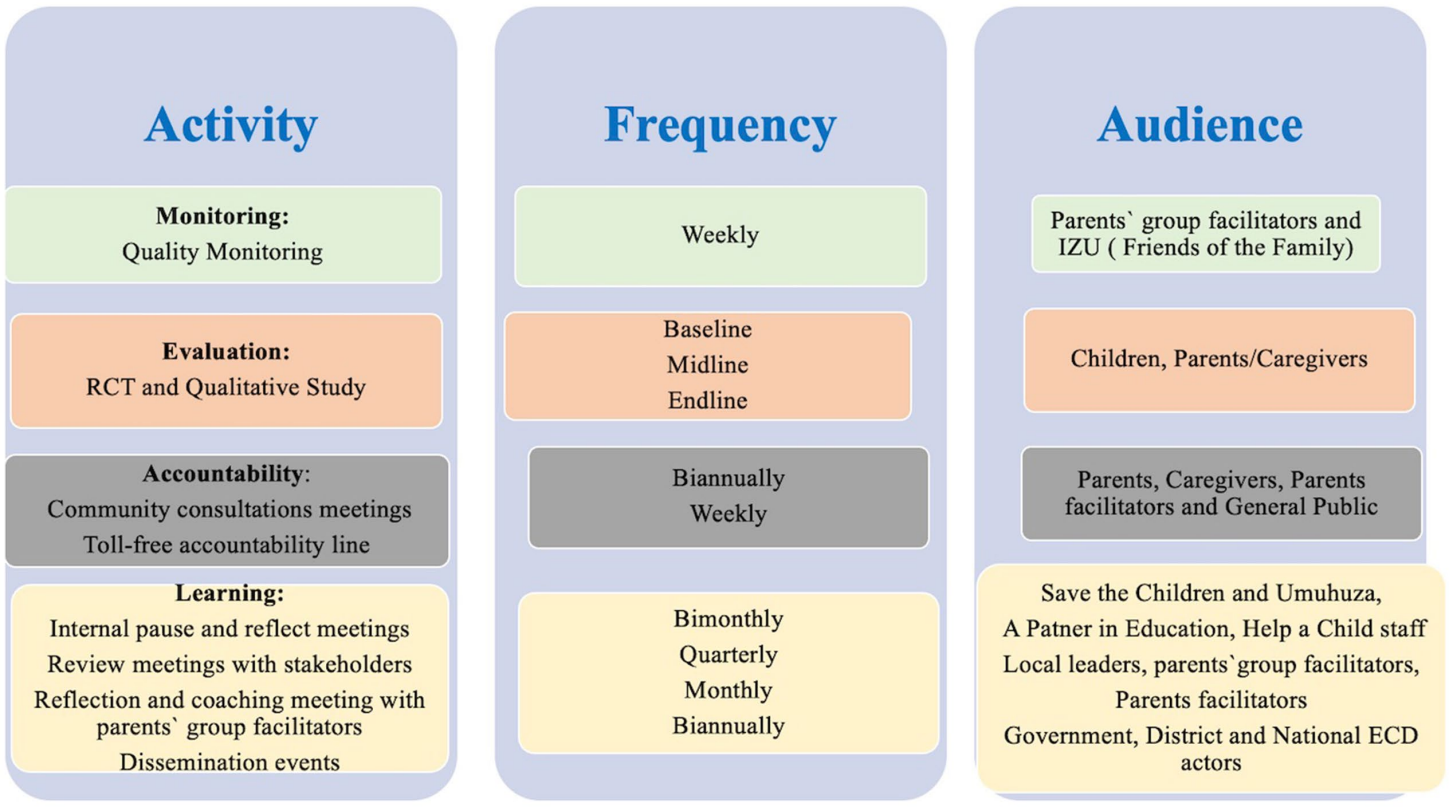
Key first steps “Intera za Mbere” monitoring, evaluation, accountability and learning (MEAL) activities.

Interruptions due to COVID-19 led to adaptations and changes to the design of the program to allow delivery amid COVID-19 meeting restrictions. COVID-19 meant that it was no longer possible for the community volunteers to convene caregivers for weekly meetings. Prior to program adaptation a COVID-19 baseline survey was conducted to understand the parenting needs, parent attitudes, and practices after the first national lockdown. The survey especially aimed to capture any impact of COVID-19 on the home environment and parents’ access to different remote delivery technology approaches. Phone-based interviews and, in some cases, face-to-face consultations were conducted with IZUs to gather their ideas on how to adapt the program to the new reality. Based on the results from this remote rapid evaluation the radio content was expanded to include a stronger emphasis on mental health and self-care, father engagement, gender, inclusion, and COVID-19 prevention and health. Radio became the primary delivery modality replacing face-to-face group meetings. The pandemic also made support to parents an even greater need than before which prompted the decision to make the radio component of the program available to all parents in Rwanda. Thus, the Radio component went to scale reaching all villages in Rwanda from October 2020. In the 3 districts of Gasabo, Ruhango, and Kirehe parents also received phone-based counseling and home visits when movement restrictions were eased. Coaching and mentoring of IZUs was also done remotely via phone. In addition to that, MoU technical partners like Help A Child encouraged and supported their beneficiaries to access and utilize the radio parenting sessions and COVID-19 parenting tips during the lockdown period. Key measurement for change activities, loops and decisions are summarized in Figures 1, 2.

### 3.5. Next steps for sustaining the intervention at scale, 2022 and beyond

Since the launch of the revised 2016 National Early Childhood Development Policy, there has been major efforts to create an enabling environment for nurturing care including the creation of the National Child Development Agency (NCDA) to oversee policy implementation and multisectoral coordination, and the expansion of IZUs terms of reference to include ECD. GoR and its partners have jointly developed the national parenting education framework. However, there is still work to be done to understand what works well in terms of building the capacity, skills, and efficacy of front-line workers such as IZUs and as well as scaling and sustaining parenting education and support for female and male caregivers/parents, as a key step to operationalizing the parenting education framework. There is a need to address gaps around ECD frontline work capacity and incentives. There is also a need to enhance the involvement of community leaders in the implementation of ECD activities at the community level. Evidence and learning from projects such as First Steps “Intera za Mbere” can bridge the gap in knowledge on how to maintain quality and fidelity of implementation at scale through existing government mechanisms.

## 4. Discussion on practical implications, lessons learned for future application

### 4.1. Embedding measurement for change principles across the project cycle

Embedding measurement for change principles and designing the project around an adaptive learning agenda allowed First Steps to have MEAL approaches owned and led by all project staff and not just the “MEAL experts.” MEAL was positioned as an integral part of the project and the responsibility of everyone including project managers, beneficiaries, partners, and all other stakeholders for a continuous quality, context-adapted, and improved implementation. We learned that for a productive and quality execution, all stakeholders need to come together through both planned and unplanned opportunities to express their views and influence decision-making across the program cycle by discussing, deciding, and working together during a needs analysis, design, implementation, monitoring, evaluation, and advocacy. Allowing for diverse voices made the program agile and responsive to the needs of stakeholders during the different changes in the implementation environment. This outlook is a necessity for similar projects wanting to make and sustain changes at scale.

### 4.2. Honoring and centering community voices

First Steps team placed an emphasis on the community voices to shape decisions and allowed the voices of parents, parent facilitators and other community members to shape the design and continuous improvement and this is one of the reasons behind the success of the approach. We learned that accountability including collecting and responding to feedback from users/beneficiaries will always be essential at all stages of the project cycle to ensure that the project implementation and design is improved and the intervention considers and accommodates the needs of its end users including their literacy levels, culture, abilities, preferences etc.

### 4.3. True and equal partnership

One of the key lessons from First Steps is the importance of working in partnership and allowing equal participation and voices of all partners. Save the Children’s implementing partner Umuhuza was involved in all decisions including on the technical content and participated in reflection and learning meetings to shape the future of the intervention. MoU technical partners such as Help a Child and A Partner in Education were also included in annual “pause, reflect, action” cycles. Quarterly reflection with the government at both national and local levels also allowed the project to respond to feedback and align with changes at policy level. We learned that Partnership is a key ingredient of a good measurement for change system.

### 4.4. Staff psychological safety and measurement for change

Our experience in embedding measurement for change principles in a project reveals that staff psychological safety is key to the fulfilling measurement for change aspirations. Staff need to be confident and free in sharing and reflecting on what is working, what is not working, what needs to change and what needs to be added to the intervention. Staff also need to be confident that this open sharing will not have negative repercussions for them, buy into the idea of measurement for change and develop a learning culture. In our case, this was encouraged through what was called the Sharing, Quality, Interactions and Discussions (SQUID), an internal Save the Children and Umuhuza mechanism for learning, reflection, and ideation. At its peak, between 2014 and 2018, staff met monthly for a SQUID meeting where learning, reflection, ideation, dreaming, reflection, and self-critique was not only encouraged but celebrated in a psychologically safe environment.

### 4.5. Combining qualitative and quantitative methodologies

A key learning from our experience is that it is important to combine both qualitative and quantitative data collection methods to inform decision-making. There is a lot of implementation science data, that can only be collected via qualitative methods, yet it is critical for capturing mechanisms of change to explain how and what is working in an intervention. Similar interventions need to embed this in design including intentionally planning stakeholder listening and reflection sessions.

### 4.6. Linking MEAL and advocacy

Our experience also taught us that it is critical to link MEAL and advocacy to achieve sustainable change. The evidence from MEAL processes should be used to continuously shape the advocacy messages over time. Evidence including from implementation is critical for providing evidence-based solutions to policy implementation gaps and in providing tools and learning on what works to inform policymakers and partner actions.

## 5. Acknowledgment of any conceptual or methodological constraints

Radio programming was one of the key elements of our program however, it was difficult to fully control contamination between intervention and control areas. For this, initially, the program was broadcast at a specific time (days and hours) which was known only to the families in the intervention group. In addition, we used the least listened-to radio station among 17 covering Ngororero district, based on our baseline assessment which successfully decreased contamination. 15% of participants in the control arm reported having listened to the radio and this spillover was controlled in the analysis. During the TtS phase in Ruhango, Gasabo and Kirehe the approach used was the same and a few families in a control group listened to one or two radio soap operas but not regularly enough to affect the results. The endline assessment showed that the intervention group had greater gains in parental practices and child outcomes.

Monitoring data also showed that some challenges were encountered in the first few weeks of implementation in 2015 but these were quickly addressed through course correction activities. For example, a few weeks after the start of the 2015 RCT, full and light interventions had a small difference due to logistical constraints in accessing books and other inputs. The importance of good logistical planning was noted for future programming and also this limitation was noted in the 2016 RCT end-line study report (5).^11^

In regards to advocacy, it is always important to recognize that advocacy is done in partnership with others so although Save the Children played an influential role in shaping policy around 0–3 ECD, it was in collaboration with other partners including the members of the Rwanda Education NGOs Coordination Platform (RENCP) ECD Working group, Imbuto Foundation, and UNICEF.

## Data availability statement

De-identified data, reports and tools supporting the conclusions of this article will be made available by the authors upon request.

## Ethics statement

The studies involving human participants were reviewed and approved by the Rwanda National Ethics Committee (RNEC). https://www.rnecrwanda.org/. Written informed consent to participate in this study was provided by the participants’ legal guardian/next of kin.

## Author contributions

CD and MA contributed to the conceptualization, design of qualitative and quantitative studies, writing, and visualization. NK and MU contributed to the review of the drafts. All authors contributed to manuscript revision, read, and approved the submitted version.

## Funding

This work was supported by the Grand Challenges Canada, Saving Brains Program, Save the Children UK (Education Breakthrough Funds), Neil Wright Foundation, and the British Academy.

## Conflict of interest

The authors declare that the research was conducted in the absence of any commercial or financial relationships that could be construed as a potential conflict of interest.

## Publisher’s note

All claims expressed in this article are solely those of the authors and do not necessarily represent those of their affiliated organizations, or those of the publisher, the editors and the reviewers. Any product that may be evaluated in this article, or claim that may be made by its manufacturer, is not guaranteed or endorsed by the publisher.

## Footnotes

^1^MIGEPROF (9) Early Childhood Development (ECD) Policy.

^2^A light touch home visit program included 2 structured home visits per family during the course of 17 week parenting program.

^3^ https://rwanda.savethechildren.net/sites/rwanda.savethechildren.net/files/library/Parenting%20Education_Literacy_Position%20Paper_2014-07-18_0.pdf

^4^The Aspirations for measurement for change (M4C) set forth by Krapels et al. (8) include recommendation on building monitoring, evaluation and learning systems that are Dynamic; Inclusive; Informative; Interactive; and People-centered.

^5^Basic package includes: parenting curriculum guide, parenting session posters, 15–20 radio sessions.

^6^Enhanced package includes: parenting curriculum guide, parenting session posters, 15–20 radio sessions+ take home cards for parents, access to a cell level book bank.

^7^Abimpaye et al. (9) improving Parenting Practices and development for young children in Rwanda: Results from a Randomized Control Trial, Int J Behav Dev. Doi: 10.1177%2F0165025419861173.

^8^IZU (Inshuti z;Umuryango or Friends of Family) is a government existing structure which is a community-based child and family protection group of volunteers composed of one man and one woman per village with main responsibilities of promoting child right, early childhood development and protection children from protection threats.

^9^ https://resourcecentre.savethechildren.net/document/common-approaches-pipeline-building-brains/

^10^ https://www.wider.unu.edu/publication/improving-parenting-practices-early-child-development

^11^ https://resourcecentre.savethechildren.net/document/saving-brains-first-steps-0-3-program-rwanda-endline-report/
